# Chewing Index: A Pilot Trial to Measure Masticatory Effort

**DOI:** 10.3390/jcm15031073

**Published:** 2026-01-29

**Authors:** Franco Marinelli, Camila Venegas-Ocampo, Josefa Alarcón-Apablaza, Rosemarie Schneider, Pablo Navarro, Ramón Fuentes

**Affiliations:** 1 Research Centre in Dental Sciences of the Universidad de la Frontera (CICO-UFRO), Facultad de Odontología, Universidad de La Frontera, Temuco 4811230, Chile; franco.marinelli@ufrontera.cl (F.M.); camilabelen.venegas@ufrontera.cl (C.V.-O.); josefa.alarcon@ufrontera.cl (J.A.-A.); r.schneider01@ufromail.cl (R.S.); pablo.navarro@ufrontera.cl (P.N.); 2 Facultad de Ciencias de la Salud, Universidad Autónoma de Chile, Temuco 4810101, Chile; 3 Núcleo de Investigación en Ciencias de la Salud, Universidad Adventista de Chile, Chillán 3780000, Chile; 4 Doctoral Program in Morphological Sciences, Faculty of Medicine, Universidad de La Frontera, Temuco 4810101, Chile

**Keywords:** functional dentition, masticatory demand, chewing muscle activity, elderly

## Abstract

**Background/Objectives:** Population aging presents new challenges for achieving healthy aging. Edentulism is a condition that diminishes quality of life. Several studies have attempted to analyze the impact of edentulism on masticatory function either by evaluating the final stage of the food bolus or the masticatory process itself. The present study aims to develop a chewing index (*Ci*) based on chewing time, the number of cycles, and the muscular activity of the masseter and temporal muscles. **Methods:** Two groups (*n* = 10 each, 60–80 years old), one with functional dentition (21 or more teeth) (Group F) and one with complete denture wearers (Group D), were used. Participants were asked to chew a total of 36 food samples. The number of chewing cycles (*N*), chewing time (*T*), and bilateral activity of the masseter and anterior temporal muscles were recorded and quantified using the root mean square. This activity was normalized with respect to a 5 s maximum voluntary clenching (*T_MAV_*). A chewing index (*Ci*) was calculated using the equation *Ci* = *N* × $\bar{V_{\%}}$ × *T*/*T_MAV_*, where $\bar{V_{\%}}$ represents the average normalized activity of the four muscles. **Results:** *Ci* values ranged from 0 to 62 for Group F and 0 to 262 for Group D. For 15 out of the 36 food samples, Ci was higher in Group D than in Group F. **Conclusions:** The results of this study are consistent with previous research showing that complete denture wearers must chew for a longer time and perform a greater number of chewing cycles compared with subjects with functional dentition.

## 1. Introduction

Population aging is a growing challenge observed across various populations worldwide [1]. As life expectancy continues to increase globally, strategies to promote healthy aging are becoming increasingly relevant [2]. In this context, oral health emerges as a relevant factor in achieving healthy aging that impacts the quality of life of older adults [3]. Tooth loss and edentulism are signs of poor oral health, which can negatively affect quality of life in older adults [4]. Edentulism negatively impacts general health, mental health, mortality [5,6], and nutrition [7], since people avoid certain foods such as fruits and meats [8,9,10,11]. There is also a psychosocial impact, as edentulous individuals are prevented from taking part in the social act of eating with their family or social circle [12,13]. The use of dental prostheses combined with dietary counseling has been associated with improvements in chewing ability, a reduced risk of cognitive impairment [14], and the prevention of malnutrition [15,16,17] in patients with tooth loss. Although it has been speculated whether prosthetic interventions alone can improve nutritional intake, the evidence remains inconclusive [14,16,18]. Recent studies suggest that these treatments should be complemented with dietary counseling in order to guide patients through the process of adapting to the prosthesis [19,20]. The use of implant-supported prostheses and mini-implants is a better option than removable dentures, although more expensive.

The methods used to assess masticatory performance include glucose extraction [21,22,23,24], chewing gum [25,26,27], and particle size distribution [28,29,30]. The glucose extraction method consists of asking the subject to chew a specially formulated gelatinous gummy for a defined period of time [22,31] or a specific number of cycles [32]. The colored mixed chewing gum method consists of providing the subject with two standardized chewing gums samples of different colors—typically green, red, or blue—to be chewed for a predetermined period of time or a specific number of cycles. Upon completion of mastication, the sample is expectorated, and the degree of color mixing is assessed either using a predefined visual scale [33,34] or through image analysis software [27]. A similar method exists that uses a single chewing gum which changes color when exposed to the acidity of saliva [25,26]. Particle size distribution involves instructing the subject to chew a standardized food item, such as peanuts [35] or Optosil [36], and to expectorate the bolus upon completion of mastication. The proportion of the sample that reaches specific particle sizes is then analyzed, either through sieving or digital image analysis [28,29,30].

Other studies evaluate chewing by recording physiological variables. Instead of asking the patient to chew a standardized food item that may be unfamiliar, commonly consumed foods such as fruits, vegetables, and meats are provided [37]. The objective is to assess whether differences emerge in the analyzed variables, including chewing time, number of cycles, and muscle activity [38,39,40,41,42,43,44,45].

These methods show wide variability in their implementation and in the comparison of results. While particle size distribution analysis depends on the type of food, the sample processing method, and the sieves used, methods such as glucose extraction or colored mixing gum require asking the subject to chew for a fixed amount of time without considering whether mastication actually fulfills its purpose—namely, preparing the bolus for swallowing. For this reason, this study presents an index derived from physiological variables recorded during the mastication of different foods, in which the subject chews until the urge to swallow is perceived, taking this as a signal that the objective of mastication has been achieved. Based on the reviewed literature, the variables chewing time, muscle activity, and the number of cycles were selected in order to design a chewing index that quantifies masticatory effort. The number of cycles was included because a subject may simply keep the food in the mouth briefly before indicating the desire to swallow. Moreover, a higher number of cycles reflects greater expressed effort. Chewing time was incorporated after observing that several studies indicate that subjects wearing complete dentures tend to chew for longer periods; therefore, chewing time may act as an adaptive mechanism. Finally, normalized muscle activity was used to incorporate a variable that is not dependent on time or on the number of cycles but rather on each subject’s maximum activation capacity. Taken together, these variables allow consideration of whether the subject actually chews, through the number of cycles; chewing time as an adaptive mechanism; and muscle activity as a measure of the individual’s level of muscular activation. The aim is to propose a control variable capable of quantifying masticatory effort and to examine whether differences exist based on the type of food and the dental status of the subjects. The hypothesis is that subjects wearing complete dentures will have a higher chewing index than those with functional dentition.

## 2. Materials and Methods

### 2.1. Statement of Ethics

The study was conducted in accordance with the Declaration of Helsinki and approved by the Ethics Committee of Universidad de La Frontera (protocol code: Nº 068_23). The volunteers gave written informed consent prior to participation.

### 2.2. Participant and Eligibility Criteria

An observational study was conducted using convenience sampling. The participants were adults aged 60 to 80 years, who were either healthy or presented with chronic systemic health conditions (such as hypertension, diabetes, or hypothyroidism) under medical control. The control group (Group F) consisted of 10 subjects with functional dentition (presence of 21 or more natural teeth, without considering the use of prostheses, according to the WHO definition [46]) whereas the intervention group (Group D) included 10 subjects with complete removable dentures.

Exclusion criteria included food allergies, hyposalivation, presence of pacemakers, implants, or prostheses in the head region that could interfere with the electromagnetic field; subjects with systemic health conditions not under medical control or with oral and/or degenerative diseases affecting mandibular movement or salivary function were excluded. Specifically, participants with neurological conditions (Parkinson’s disease, Alzheimer’s disease, senile dementia) or conditions/medications known to impair salivary flow (Sjögren’s syndrome, head and neck radiation therapy, medications with xerostomia as a side effect) were excluded, verified through medical history review and clinical examination. Clinical temporomandibular dysfunction was assessed and excluded based on absence of joint sounds or clicking during lateral and protrusive movements, absence of pain on palpation of the temporomandibular joints and masticatory muscles, and absence of pain or difficulty during mastication. Additionally, subjects with restricted mandibular mobility or musculoskeletal pain affecting mastication were excluded. A symptom questionnaire for TMD (Spanish version of the tool developed by the International Network for Orofacial Pain and Related Disorders Methodology) [47] was applied by a trained dentist (R.S.) at the Oral Physiology Laboratory of Universidad de La Frontera.

An unstimulated and stimulated salivary flow test was performed according to Neyraud et al. [48] in order to exclude subjects with hyposalivation that could impair food mastication. A salivary flow rate greater than 0.1 mL/min for the unstimulated test and greater than 0.5 mL/min for the stimulated test was considered a normal value.

All exclusion criteria were determined through comprehensive clinical examination and patient interview.

Demographic and clinical information was recorded for all participants, including age, sex, and, for Group D, the duration of current complete removable denture use. However, information regarding total lifetime denture-wearing experience was not systematically assessed, which represents a methodological limitation discussed below.

### 2.3. Variables

The primary independent variable was dental status (dentate subjects with functional dentition versus complete denture wearers). The secondary independent variable was food type (36 food samples varying in texture and hardness). Dependent variables included the chewing index (*Ci*) and its component physiological parameters: chewing duration (T in seconds), the number of chewing cycles (*N*), and normalized muscle activity ($\bar{V_{\%}}$). Potential confounding variables such as facial biotype, total lifetime denture-wearing experience, and neuromuscular adaptation. All measurements were obtained through electromagnetic articulography, surface electromyography, and clinical assessment, as detailed below.

### 2.4. Movement Recording Techinc

Electromagnetic articulography (EMA) is a technique based on the principle of magnetic induction. The system comprises three emitter coils that generate variable electromagnetic fields in the range of 7–12 kHz and miniature receiver coils (2 × 2 mm) (AG501, Carstens Medizinelektronik, Bovenden, Germany) that act as motion detectors. The induced currents are used to determine the precise spatial position of the receiver coils, functioning as motion sensors. The EMA system has a spatial accuracy of approximately 0.3 mm and has been validated as a reliable method for analyzing mandibular and chewing movements [49]. Due to this procedure, patients with metallic elements near the head had to be excluded. In this study, the number of chewing cycles and the chewing time (in seconds) were evaluated at the Oral Physiology Laboratory of the Universidad de La Frontera (Temuco, Chile) using a 3D electromagnetic articulograph (AG501, Carstens Medizinelektronik, Bovenden, Germany).

Seven pre-calibrated EMA sensors were used to record chewing cycles and chewing time: one active sensor, three reference sensors, and three attached to the bite-plane accessory. In addition, the articulograph was fitted with a grounded connector placed on the participant’s wrist. The active sensor was positioned at the interincisal midline—either on the natural teeth (Group F) or on the complete denture (Group D)—while the reference sensors were attached to the participant’s head. These reference sensors provided a baseline for correcting head motion and standardizing the recording of mandibular movement by the Head Correction procedure [11]. The bite-plane accessory of the articulograph was used to define the coordinate origin in the occlusal plane. If necessary, the Head Correction procedure was repeated; this may have been due to the movement of the reference sensors.

### 2.5. Surface Electromiographgy

Electromyographic recording was performed using surface electromyography (EMG VIII; ArtOficio, Santiago de Chile, Chile) with a sampling frequency of 1311 Hz, CMRR of 110 dB, and input impedance of 10 Gohm. A band-pass filter (10–500 Hz) was used. Bipolar Ag/AgCl electrodes with an interelectrode distance of 24 mm and a conductive area of 80 mm^2^ (H124SG, Kendall, Waukegan, IL, USA) were used. The muscular activity of the right and left anterior temporalis and masseter muscles was recorded. The electrode placement and signal filtering procedures were the same as those used in a previous study [50]. The skin was previously cleaned with gauze soaked in 70% alcohol. The electrodes were placed on the belly of the muscle, which was located by palpation while the subject performed a jaw clenching. The electrodes were placed parallel to the direction of the muscle fibers, and a reference electrode was attached to the participant’s elbow. The records were performed between 9 a.m. and 4 p.m.

### 2.6. Chewing Records

Thirty-six foods (Table 1) were tested in order to analyze chewing behavior for both groups. To avoid patient fatigue due to the length of the recording sessions, the foods were divided into two separate sessions.

Once the AG501 3D EMA sensors (AG501, Carstens Medizinelektronik, Bovenden, Germany) and EMG electrodes (H124SG, Kendall, Waukegan, IL, USA) were in place, each participant was instructed to remain seated with their back straight and the Frankfort plane parallel to the floor. They were asked to chew until they felt the need to swallow but without actually doing so. Upon feeling the urge to swallow, they were instructed to raise their left hand as a signal to indicate the end of mastication. Each participant was given a sample of each food and asked to hold it between the tongue and the palate until receiving the signal to begin chewing. The end of the chewing cycle marked the end of the recording. One recording was performed per food item. To quantify the maximum capacity of the masticatory muscles, three 5 s recordings of maximum voluntary clenching were performed with two minutes of rest between them. The root mean square (RMS) values were calculated, and the highest value recorded was used as the reference.

### 2.7. Recording Synchronization

Muscle activity and mandibular movement recordings were synchronized using the method described by Lezcano et al. [51].

### 2.8. Chewing Index

Fan et al. [52] and Gonçalves et al. [53] define three types of assessments, objective (direct and indirect) and subjective. Direct objective methods are those that analyze the effect of chewing on a test food. The number of cycles the subject must perform is usually defined, or the subject is asked to chew until they feel the need to swallow. Indirect objective methods analyze kinematic and physiological variables such as chewing time, muscle activity, the number of cycles, frequency, etc. Finally, subjective methods are based on questionnaires in which the patient defines the perceived difficulty of chewing.

The mastication index falls within the indirect methods. Instead of analyzing the various characteristics of chewing, we propose designing an indicator that defines masticatory effort. For this purpose, the mastication index is defined based on the following equation:

(1) $$Ci=N\times \bar{V_{\%}}\times \frac{T}{T_{MAV}}$$

where

*N* is the number of chewing cycles;

T is the chewing time in seconds;

T*_MAV_* is the time of the maximum voluntary clenching record, which for all cases is 5 s.

$\bar{V_{\%}}$ is the average of the normalized root mean square (RMS) value of the left and right masseter and anterior temporalis muscles. This value was obtained using the following formula:

$$\bar{V_{\%}}=\frac{1}{4}\sum_{i=1}^{4}\frac{V_{RMSi}}{V_{MVCi}}$$

where

*V_RMSi_* is the root mean square value for the muscle *i* (right anterior temporal, left anterior temporal, right masseter, and left masseter) during chewing;

*V_MVCi_* is the root mean square value for the muscle *i* (right anterior temporal, left anterior temporal, right masseter, and left masseter) during maximum voluntary clenching.

When the subject does not perform any chewing, *N* is 0, and therefore *Ci* is also 0.

The calculation is proposed considering that the greater the chewing difficulty is, the longer the food will be chewed and the higher the number of chewing cycles will be. Muscle activity reflects the effort the subject exerts to break down the food, although it is not a direct measurement of that effort [54]. Since each subject exhibits different muscle activity levels, a normalization process was performed using the maximum voluntary clench. Time was also normalized so that the index would be expressed in dimensionless units or number of cycles.

The data were processed using Matlab routines (2020a, Version: 9.8.0.1323502; The MathWorks Inc., Natick, MA, USA). The time and number of cycles were extracted from the EMA recording and the muscle activity from the electromyographic recording. The number of cycles was established by determining the sensor’s position relative to the position reached during closing (P0). A range of 3 mm below the closing position, on the vertical axis, was used as the start/end indicator. While the sensor was within the range P0–P0 + 3 mm, the midpoint was located and used as the start/end of each cycle.

### 2.9. Statistical Analisys

Normality was assessed using the D’Agostino test. For group comparisons, the independent-sample *t*-test and the Mann–Whitney U test were applied. Data analysis was performed using SPSS Statistics for Windows (version 23.0, IBM, Armonk, NY, USA). A *p*-value < 0.05 was considered the threshold for statistical significance. Appendix A shows the mean differences, confidence intervals, and effect sizes for each variable. Effect sizes were categorized according to Zieliński et al. [55]. Due to the length of these tables, they are not included in the main manuscript.

## 3. Results

### 3.1. Participant Information

The sample consisted of 20 participants (10 men and 10 women) divided into two groups: Group F (functional dentition) with 10 participants (5 men, 5 women; mean age = 61.3 ± 1.06 years) and Group D (complete denture wearers) with 10 participants (5 men, 5 women; mean age = 68.6 ± 7.0 years). Group F presented a mean of 24.2 ± 3.5 natural teeth, meeting the criterion of ≥21 teeth for functional dentition.

Clinical and Health Characteristics:

Group F: Seven participants (70%) reported at least one controlled chronic condition, including diabetes, hypothyroidism, hypertension, and Crohn’s disease. Mean unstimulated salivary flow was 0.238 ± 0.126 mL/min and mean stimulated salivary flow was 1.094 ± 0.654 mL/min.
Group D: Eight participants (80%) reported at least one controlled chronic condition, including hypertension, diabetes, and asthma with pre-diabetes. Mean unstimulated salivary flow was 0.400 ± 0.362 mL/min and mean stimulated salivary flow was 1.257 ± 0.691 mL/min.

All salivary flow measurements met the inclusion criteria for normal salivary flow (unstimulated: >0.1 mL/min; stimulated: >0.5 mL/min).

Regarding denture tenure in Group D, the duration of current denture use ranged from 6 months to 10 years (mean = 3.92 ± 3.5 years).

### 3.2. Chewing Index Values by Food Category

**Figure 1:**
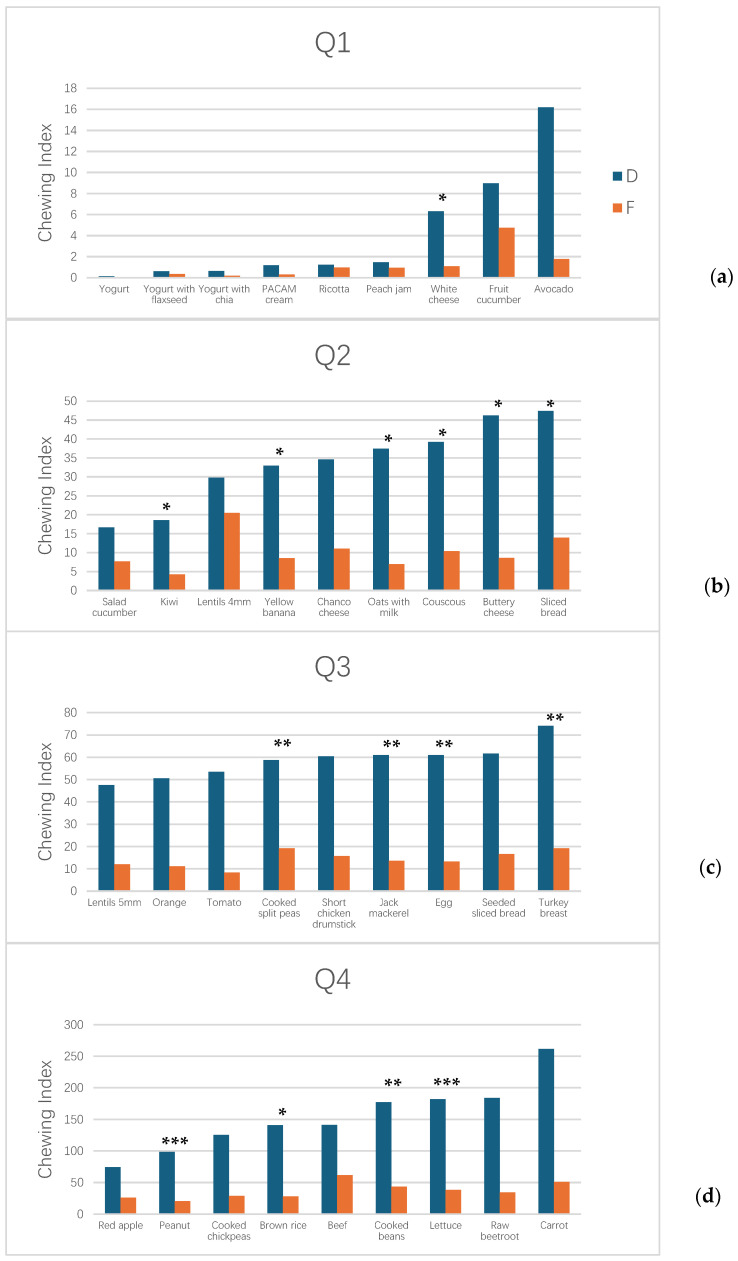
Chewing index for functional dentition (F) and complete denture wearers (D). (**a**) First quartile, values from 0.1 to 16. (**b**) Second quartile, values from 17 to 47. (**c**) Third quartile, values from 47 to 74. (**d**) Fourth quartile, values from 75 to 261. (* *p* < 0.05; ** *p* < 0.01; *** *p* < 0.001).

The *Ci* values demonstrated substantial variation across the 36 food samples, with notable differences between groups. Figure 1 presents *Ci* values stratified by quartiles to facilitate comparison across the range of food hardness and texture. *Ci* values for Group F ranged from 0.0 to 51.0, while those for Group D ranged from 0.0 to 262, representing a tenfold difference in maximum *Ci* values between groups. For 15 of the 36 food samples tested, *Ci* was significantly higher in Group D than in Group F (*p* < 0.05).

In the first quartile (Q1, Figure 1a), comprising soft and semi-solid foods (yogurt, yogurt with flaxseed, yogurt with chia, PACAM cream, ricotta, peach jam, white cheese, fruit cucumber, and avocado), Group F exhibited *Ci* values from 0.0 to 1.8, while Group D showed higher values ranging from 0.0 to 16.2.

The second quartile (Q2, Figure 1b), containing foods of intermediate hardness (salad cucumber, kiwi, lentils, yellow banana, Chanco cheese, oats with milk, couscous, buttery cheese, and sliced bread), showed an intermediate pattern, with Group F *Ci* values ranging from 7.7 to 13.9 and Group D values from 16.7 to 47.4.

The third and fourth quartiles (Q3 and Q4, Figure 1c,d) included harder and more fibrous foods (5 mm lentils, orange, tomato, cooked split peas, chicken drumstick, jack mackerel, egg, seeded sliced bread, turkey breast, red apple, peanut, cooked chickpeas, brown rice, beef, cooked beans, lettuce, raw beetroot, and carrot). In these categories, the separation between groups became most pronounced, with Group D exhibiting *Ci* values substantially exceeding those of Group F across nearly all food items.

### 3.3. Analysis of Component Variables

Table 2 presents the mean values of chewing time (*T*), the number of chewing cycles (*N*), muscle activity ($\bar{V_{\%}}$), and the chewing index (*Ci*) for all 36 food samples across both groups. Overall, Group D exhibited significantly longer chewing times and greater numbers of cycles compared to Group F across most food items. Specifically, six foods showed significant differences in chewing time between groups (all with longer durations in Group D: jack mackerel, turkey breast, lettuce, carrot, and others), while six foods showed significant differences in cycle number (all with higher cycles in Group D). Twenty-two foods demonstrated significant differences in relative muscle activity, with Group D consistently showing higher normalized activity levels across food categories (*p* < 0.05).

*Ci* revealed significant differences between groups for 15 foods (all with higher values in Group D), spanning across all quartile categories. The highest *Ci* values were observed in harder foods (Q4): carrot (*Ci* = 262 for Group D vs. 51 for Group F), cooked beans (177 vs. 43.4), and lettuce (182 vs. 38.4). Conversely, soft and semi-solid foods (Q1) showed the lowest *Ci* values in both groups, with yogurt producing *Ci* values of 0.15 for Group F and 0.3 for Group D.

## 4. Discussion

Six foods showed significant differences between groups based on chewing time always with a longer chewing time for Group D. There were six foods that showed significant differences between Groups F and D. In all cases, Group D exhibited a higher number of cycles. Of these six foods, four also showed differences in chewing time (jack mackerel, turkey breast, lettuce, and carrot). This has been observed in previous studies [40,44,56,57,58]. Kohyama et al. [40] found that older adults with poorer dental status had longer chewing times when chewing bread, apple, and peanuts compared with those with better dental status and a young control group. Huang et al. [56] found that subjects with two or fewer posterior occlusal pairs in the support zone, according to the Eichner classification, chewed for a longer time compared with those who had four occlusal pairs. Oncescu et al. found that complete denture wearers chewed biscuits, carrots, and apples for a longer time and a greater number of cycles than partial denture wearers.

When analyzing relative muscle activity, twenty-two foods showed significant differences between Groups F and D. In all cases, Group D exhibited higher relative muscle activity. This contradicts other studies reporting that the muscle activity of fully dentate subjects is greater than that of denture wearers [44,57]. However, this can be explained because what is reported here is the muscle activity relative to that obtained during maximum voluntary clenching. Neves et al. [59] found that muscle activity during maximum voluntary clenching and chewing was lower in complete denture wearers than in subjects with natural dentition. They explain this by the tendency to perform bilateral mastication in order to stabilize the prosthesis and to avoid damaging it. These factors should be considered when evaluating masticatory effort. Slagter et al. [60] found results similar to those of this study when normalizing muscle activity during chewing with respect to maximum voluntary clenching. Von Der Gratch et al. [60] found that muscle activity is lower during maximum voluntary clenching and is also lower during mastication, although it depends on the hardness of the foods. Park et al. [44] found lower muscle activity during mastication in complete denture wearers in the masseter muscles but not in the temporalis muscles. Kohyama et al. [40] found lower muscle activity during maximum voluntary clenching but did not find differences in muscle activity during mastication. The literature suggests that complete denture wearers usually have lower muscle activity during maximum voluntary clenching and similar activity during mastication. Based on this, it is expected that the relative activity of complete denture wearers would be higher, as our results show.

Kohyama et al. [40] found that subjects with low dental status tend to have less muscle activation but a higher number of cycles, which provide constant work to grind the food. Although this inverse relationship—where muscle activity decreases while the number of cycles increases—appears to be compensated, Witter et al. [58] found that subjects wearing complete dentures have a masticatory efficiency 50% lower than those with a full natural dentition.

Analysis of the literature indicates that subjects wearing complete dentures tend to chew a greater number of times but with lower or similar muscle activity than subjects with natural dentition. They also tend to exhibit lower muscle activity during maximum voluntary clenching; therefore, although their absolute activation is lower, it is relatively higher when considered in relation to their maximal capacity. Normalization based on maximum voluntary clenching may not be the most appropriate method, since subjects wearing complete dentures may not exert their true maximum effort during maximum voluntary clenching due to fear of damaging the prosthesis or its instability. Finally, masticatory efficiency has been found to be lower in subjects wearing complete dentures, despite compensating for their reduced muscle activity during mastication by chewing a greater number of times. The tendency of subjects wearing complete dentures to chew for a longer time and a greater number of times and the use of normalization based on maximum voluntary clenching explain why the chewing index tends to be higher for this group.

When analyzing the chewing index (Table 2), we found 15 foods (white cheese, kiwi, yellow banana, oats with milk, couscous, buttery cheese, sliced bread, cooked split peas, jack mackerel, egg, turkey breast, peanut, brown rice, cooked beans, and lettuce) with significant differences between Groups F and D, always with a higher value for Group D. This number is higher than that when comparing by time or number of cycles (6 for both) but lower than that in the case of muscle activity where 22 foods showed significant differences. This variability may be due to the multiplicity of factors that affect mastication, such as prosthesis stability, food texture, and the subject’s familiarity with the food [59]. In a previous study [61], the hardness of the foods used was evaluated. As seen in Figure 1a, fluid or semi-solid foods showed a lower chewing index. On the other hand, foods with higher hardness showed a higher chewing index (Figure 1d), such as beef, carrot, raw beet, or cooked beans. The functional dentition group showed a narrower range of the chewing index, reaching a maximum of 60 for beef (Figure 1d). In contrast, the group of subjects with complete dentures showed a continuous increase. This can be seen in Figure 1a,b,d. In the third quartile in Figure 1c, some stability is observed, with values between 47 and 74.

If we mathematically analyze how the chewing index is composed (Equation (1)), we can note that time and the number of cycles are related, since it is expected that a higher number of cycles would require more time at a constant frequency. Uram-Tuculescu et al. [62] found no significant differences in chewing frequency between complete denture wearers and subjects with 10 or more occlusal pairs. Mishellany-Dutour et al. [63] found that chewing frequency was lower in adults wearing complete dentures. These studies show that chewing frequency varies within a narrow range, between 1.5 and 1.8 cycles per second. If we consider chewing frequency (*f*) as *N*/*T*, we can rewrite Equation (1) as

(2) $$Ci=N\times \bar{V_{\%}}\times \frac{T}{T_{MAV}}=N\times \bar{V_{\%}}\times \frac{T}{T_{MAV}}\times \frac{T}{T}=f\times \bar{V_{\%}}\times \frac{T^{2}}{T_{MAV}}$$

Or:

(3) $$Ci=N\times \bar{V_{\%}}\times \frac{T}{T_{MAV}}=N\times \bar{V_{\%}}\times \frac{T}{T_{MAV}}\times \frac{N}{N}=\bar{V_{\%}}\times \frac{N^{2}}{f\times T_{MAV}}$$

Given that *T_MAV_* is constant, *V_%_* varies between 0 and 1 (ideally), and frequency is also limited, we can observe that time and the number of cycles are the variables that most influence the final *Ci* value, as it varies quadratically with these, while it varies linearly with muscle activity. This may explain why *Ci* shows differences in a smaller number of foods than muscle activity, since the latter exhibits its variability linearly, but greater differences than when evaluating time and the number of chewing cycles individually. An interesting aspect is that in the study of masticatory performance, the number (*N*) and/or time of mastication (*T*) is fixed [64,65]; therefore the chewing index would be defined as follows:

For constant *T*,

(4) $$Ci=f\times \bar{V_{\%}}\times \frac{T^{2}}{T_{MAV}}=f\times \bar{V_{\%}}\times C$$

For constant *N*,

(5) $$Ci=\bar{V_{\%}}\times \frac{N^{2}}{f\times T_{MAV}}=\frac{\bar{V_{\%}}}{f}\times C$$

The development of a chewing index addresses an important gap in clinically relevant assessment of total removable denture function. *Ci* offers several advantages over current chairside or research-based masticatory assessment tools. Compared to color-mixing gum indices, *Ci* provides objective, quantitative measurements of physiological variables rather than relying on subjective visual grading scales, potentially reducing inter-observer variability. Relative to sieving methods, *Ci* is less uncomfortable for elderly patients with dentures, as it requires no expectoration and utilizes real foods that patients consume regularly. In contrast to patient-reported chewing indices, *Ci* is independent of subjective perception and directly captures actual neuromuscular compensation strategies that patients may not consciously recognize. While bite force devices measure only maximal force generation, *Ci* integrates multiple physiological dimensions—chewing duration, the number of cycles, and normalized muscle activity—thereby capturing the multifactorial nature of masticatory demand and compensation.

The potential clinical applications of *Ci* extend beyond research settings. Such an index could serve as a longitudinal monitoring tool for evaluating denture efficacy, tracking neuromuscular adaptation to new prostheses over time, and comparing masticatory outcomes between different rehabilitative approaches (removable dentures versus implant-supported prostheses). By integrating multiple physiological variables, this index would capture the multifactorial nature of masticatory compensation better than single-variable measures such as bite force devices or subjective patient-reported indices. This study provides preliminary evidence and generates hypotheses for future confirmatory research; a formal assessment of reliability and validity is necessary. Specifically, test–retest and inter-observer correlations with other established masticatory performance indices and a formal validation against gold-standard measures are needed.

Limitations of this study are the small number of subjects, especially for studies related to the activity of the masticatory muscles [66]. An inter-subject analysis could not be performed because each chewing task was carried out only once. This was due to the length of the recording sessions resulting from the number of foods used. Although the normalization method using maximum voluntary clenching has been employed in previous studies, it is necessary to analyze whether this is the most appropriate for subjects with other conditions, such as temporomandibular disorders, since these individuals may be limited in performing maximum effort. In the case of subjects wearing dentures, they may not exert their maximum effort due to fear of damaging the prosthesis. Alternative normalization methods should be considered [67]. Chewing can be affected by salivary flow [68], sarcopenia [25], the number of teeth [21], food texture, and the subject’s familiarity with the food [59]. While hyposalivation was considered an exclusion criterion, hypersalivation was not considered a confounding factor. Only a number of teeth greater than 21 was considered for the functional group, and the presence of sarcopenia was not determined. The skeletal class of the subjects was also not considered. In the case of subjects wearing complete dentures, no analysis of the level of prosthesis fixation was performed. No comparisons were made between foods within the same group. The equipment used is complex and limited to research settings. The number of chewing cycles and chewing time could be determined using video recordings, but muscle activity requires not only specialized equipment but also trained personnel.

A significant limitation regarding denture adaptation is that while we assessed the duration of current complete denture use (6 months to 10 years), neuromuscular adaptation to dentures likely accumulates over total cumulative use rather than duration of the current prosthesis alone. Participants may have replaced their dentures at various points throughout their lives, and a recently fabricated denture does not necessarily indicate limited overall adaptation. Conversely, a longer interval since current denture insertion does not necessarily reflect greater cumulative adaptation experience. This limitation prevents definitive assessment of whether elevated *Ci* values in denture wearers reflect biomechanical constraints of the prosthesis or residual neuromuscular adaptation from prior denture experience. Future studies should systematically collect both current denture duration and total lifetime denture-wearing history and employ stratified or adjusted analyses to control for cumulative adaptation effects.

An important limitation regarding the interpretation of *Ci* is that it may conflate masticatory inefficiency with biological effort. While *Ci* effectively quantifies the physiological demand required to process food, higher *Ci* values in complete denture wearers should be interpreted as reflecting masticatory inefficiency and adaptive compensation rather than greater muscular effort.

## 5. Conclusions

The present study evaluated mastication based on physiological parameters such as chewing time, the number of cycles, and normalized muscle activity. The results obtained are consistent with the consulted literature. A chewing index was designed to assess the effort required by subjects to form the food bolus. Higher values were observed for complete denture wearers. This study is limited by a small sample size and to the performance of a single chewing trial per food; therefore, studies with a larger number of subjects are needed and to evaluate inter-subject variability. *Ci* could be used in research involving the study of masticatory performance where the number of cycles or the time is fixed. Further studies are required that include frequency as a variable of analysis during mastication in complete denture wearers. This study provides preliminary evidence and generates hypotheses for future confirmatory research.

## Figures and Tables

**Table 1:** Food list for chewing records.

Session 1			Session 2				
Fruits	Dairy	Vegetables	Vegetal Protein	Animal Protein	Cereals and Grains	High-Lipid Food and Others	
Kiwi	Soft unsalted cheese	Tomato	Lentils, 4 mm	Eggs	Oatmeal and milk	Yogurt and flax	Peanut
Orange	Chanco cheese	Lettuce	Lentils, 5 mm	Jack mackerel	Whole-wheat sliced bread	Yogurt and chia	
Banana	Buttery cheese	Cucumber	Beans	Chicken thigh	Whole-wheat bread with seeds	Peach jam	
Sweet cucumber	Yogurt	Raw carrot	Chickpeas	Turkey breast	Whole-grain rice	PACAM cream	
Red apple	Ricotta	Raw beets	Split peas	Beef	Couscous	Avocado	

**Table 2:** Average value of variables that make up the chewing index: chewing time, muscle activity, and number of cycles.

Quartile	Foods	Time [s]			*N*			$\bar{V_{\%}}$			*Ci*		
		F	D	*p*	F	D	*p*	F	D	*p*	F	D	*p*
Q1	Yogurt	0.3	1.2	n.s.	0.3	0.9	n.s.	0.1	0.3	0.01 *	0.01	0.15	n.s.
	Yogurt with flaxseed	2.7	1.8	n.s.	1.9	2	n.s.	0.2	0.3	0.03 *	0.36	0.63	n.s.
	Yogurt with chia	2.4	1.8	n.s.	2	2.1	n.s.	0.1	0.3	0.002 *	0.19	0.64	n.s.
	PACAM cream	2.4	2.6	n.s.	2.5	2.6	n.s.	0.2	0.3	0.03 *	0.31	1.19	n.s.
	Ricotta	4.4	2.9	n.s.	3.9	2.8	n.s.	0.2	0.3	n.s.	0.98	1.24	n.s.
	Peach jam	3.8	2.9	n.s.	4.8	3.6	n.s.	0.2	0.3	0.04 *	0.95	1.47	n.s.
	White cheese	5.2	7.2	n.s.	5.8	8.3	n.s.	0.2	0.4	<0.001 *	1.1	6.3	0.02 *
	Fruit cucumber	8.2	9.3	n.s.	12	11.1	n.s.	0.2	0.4	0.004 *	4.7	8.9	n.s.
	Avocado	5.4	10.2	n.s.	6.6	13.1	0.04 *	0.2	0.4	0.005 *	1.77	16.2	n.s.
Q2	Salad cucumber	8	10.6	n.s.	13	14.5	n.s.	0.4	0.5	n.s.	7.67	16.6	n.s.
	Kiwi	8.8	12.5	n.s.	11	14.1	n.s.	0.3	0.5	0.01 *	4.3	18.6	0.01 *
	Lentils, 4 mm	13	15.2	n.s.	19	18.3	n.s.	0.4	0.5	0.046 *	20.5	29.7	n.s.
	Yellow banana	11	16.6	n.s.	14	17.4	n.s.	0.3	0.5	0.01 *	8.5	33.1	0.01 *
	Chanco cheese	12	16.7	n.s.	15	17.5	n.s.	0.3	0.5	0.03 *	11	34.6	n.s.
	Oats with milk	8.6	15.2	n.s.	9.8	16.3	n.s.	0.4	0.4	n.s.	7	37.5	0.04 *
	Couscous	12	16.8	n.s.	15	19.5	n.s.	0.3	0.4	0.01 *	10.4	39.2	0.04 *
	Buttery cheese	11	19	n.s.	13	21	n.s.	0.3	0.5	n.s.	8.6	46.2	0.03 *
	Sliced bread	13	19.2	n.s.	16	21.5	n.s.	0.3	0.5	0.01 *	13.9	47.4	0.04 *
Q3	Lentils, 5 mm	9.9	17.2	n.s.	14	20.7	n.s.	0.4	0.5	0.02 *	12	47.5	n.s.
	Orange	9.3	17.5	n.s.	12	18.6	n.s.	0.4	0.6	n.s.	11.2	50.5	n.s.
	Tomato	9.3	16.9	n.s.	13	20.8	n.s.	0.3	0.6	0.007 *	8.4	53.5	n.s.
	Cooked split peas	13	20.3	n.s.	19	27.4	n.s.	0.3	0.5	0.01 *	19.2	58.7	0.01 *
	Short chicken drumstick	13	19.5	n.s.	17	24.3	n.s.	0.4	0.4	n.s.	15.7	60.4	n.s.
	Jack mackerel	11	21.3	0.02 *	15	25.1	0.02 *	0.4	0.5	n.s.	13.6	61	0.01 *
	Egg	14	22.1	0.03 *	19	24.9	n.s.	0.3	0.5	0.01 *	13.3	61	0.004 *
	Seeded sliced bread	14	21.1	n.s.	18	23.9	n.s.	0.3	0.4	0.007 *	16.7	61.7	n.s.
	Turkey breast	14	24.9	0.03 *	18	29.3	0.04 *	0.4	0.5	n.s.	19.2	74.1	0.002 *
Q4	Red apple	13	20.6	n.s.	18	25.1	n.s.	0.4	0.6	n.s.	25.8	74.2	n.s.
	Peanut	11	24.5	n.s.	16	30.8	0.02 *	0.5	0.6	n.s.	20.2	98.4	0.001 *
	Cooked chickpeas	16	28	n.s.	22	35.3	n.s.	0.4	0.5 *	0.04 *	28.9	125	n.s.
	Brown rice	17	29.9	0.04 *	22	34.9	n.s.	0.4	0.5	0.01 *	27.7	141	0.48 *
	Beef	21	30	n.s.	27	35.4	n.s.	0.5	0.6	n.s.	61.6	141	n.s.
	Cooked beans	18	33.6	n.s.	25	40.1	0.04 *	0.5	0.6	0.04 *	43.4	177	0.01 *
	Lettuce	17	33.8	<0.001 *	23	39.9	0.007 *	0.5	0.7	n.s.	38.4	182	<0.001 *
	Raw beetroot	15	21.2	n.s.	23	25.8	n.s.	0.5	0.8	n.s.	34.1	184	n.s.
	Carrot	15	35.2	0.007 *	25	43.5	0.02 *	0.5	0.7	n.s.	51	262	n.s.

F: functional dentition group, D: complete denture wearer group, T: chewing time in seconds, *N*: number of chewing cycles, $\bar{V_{\%}}$: average normalized muscle activity, *Ci*: chewing index, n.s.: no significance; * *p* < 0.05, indicating significant differences.

## Data Availability

The data presented in this study are available on request from the corresponding author. The data are not publicly available since no public database is available.

## Appendix A

**Table A1:** Mean differences, confidence intervals, and effect sizes (Hedges’ g) for chewing time between the functional dentition (F) group and the complete denture wearer (D) group.

Quartile	Foods	Time [s]			
		F − D	CI	g	Effect Size
Q1	Yogurt	−0.94	(−2.20, 0.36)	−0.7	Large
	Yogurt with flaxseed	0.86	(−2.20, 3.95)	0.3	Medium
	Yogurt with chia	0.57	(−1.75, 2.88)	0.2	Small
	PACAM cream	−0.18	(−3.53, 3.18)	−0.04	Small
	Ricotta	1.44	(−1.62, 4.50)	0.4	Medium
	Peach jam	0.84	(−1.86, 2.89)	0.3	Medium
	White cheese	−1.94	(−6.01, 2.13)	−0.4	Medium
	Fruit cucumber	−1.12	(−5.70, 3.45)	−0.2	Small
	Avocado	−4.77	(−10.7, 11.1)	−0.7	Large
Q2	Salad cucumber	−2.54	(−7.98, 2.89)	−0.4	Medium
	Kiwi	−3.70	(−8.50, 1.10)	−0.7	Large
	Lentils, 4 mm	−2.20	(−6.67, 2.29)	−0.4	Medium
	Yellow banana	−5.66	(−11.57, 0.26)	−0.9	Large
	Chanco cheese	−4.43	(−10.94, 2.07)	−0.6	Medium
	Oats with milk	−6.67	(−16.40, 3.06)	−0.6	Medium
	Couscous	−4.77	(−13.53, 3.98)	−0.5	Medium
	Buttery cheese	−8.48	(−18.22, 1.24)	−0.8	Large
	Sliced bread	−6.42	(−16.09, 3.25)	−0.6	Medium
Q3	Lentils, 5 mm	−7.26	(−12.48, −2.04)	−1.3	Large
	Orange	−8.18	(−16.48, 0.11)	−0.9	Large
	Tomato	−7.61	(−18.68, 3.45)	−0.6	Medium
	Cooked split peas	−7.79	(−13.91, −1.67)	−1.1	Large
	Short chicken drumstick	−6.96	(−17.36, 3.45)	−0.6	Medium
	Jack mackerel	−10.03	(−18.23, −1.83)	−1.1	Large
	Egg	−8.24	(−17.51, 1.03)	−0.8	Large
	Seeded sliced bread	−7.60	(−20.20, 4.97)	−0.5	Medium
	Turkey breast	−10.98	(−19.10, −2.86)	−1.2	Large
Q4	Red apple	−8.05	(−20.71, 4.62)	−0.6	Medium
	Peanut	−13.25	(−19.13, −7.37)	−2.1	Large
	Cooked chickpeas	−12.05	(−23.47, −0.62)	−0.9	Large
	Brown rice	−13.36	(−25.04, −1.68)	−1.0	Large
	Beef	−9.00	(−19.61, 1.61)	−0.8	Large
	Cooked beans	−15.43	(−25.99, −4.86)	−1.3	Large
	Lettuce	−17.32	(−27.61, −7.02)	−1.5	Large
	Raw beetroot	−6.24	(−17.94, 5.44)	−0.5	Medium
	Carrot	−19.84	(−42.32, 2.65)	−0.8	Large

**Table A2:** Mean differences, confidence intervals, and effect sizes (Hedges’ g) for the number of chewing cycles (N) between the functional dentition (F) group and the complete denture wearer (D) group.

Quartile	Foods	N			
		F − D	CI	g	Effect Size
Q1	Yogurt	−0.60	(−1.56, 0.57)	−0.5	Medium
	Yogurt with flaxseed	−0.10	(−2.74, 2.54)	−0.03	Small
	Yogurt with chia	−0.10	(−2.71, 2.51)	−0.03	Small
	PACAM cream	−0.10	(−3.27, 3.07)	−0.03	Small
	Ricotta	1.10	(−1.86, 4.06)	0.3	Medium
	Peach jam	1.20	(−2.21, 4.61)	0.3	Medium
	White cheese	−2.50	(−7.52, 2.52)	−0.5	Medium
	Fruit cucumber	0.80	(−5.05, 6.65)	0.1	Small
	Avocado	−6.50	(−13.30, 0.30)	−0.9	Large
Q2	Salad cucumber	−1.50	(−9.12, 6.12)	−0.2	Small
	Kiwi	−2.70	(−8.47, 3.07)	−0.4	Medium
	Lentils, 4 mm	0.30	(−7.76, 8.37)	0.03	Small
	Yellow banana	−3.70	(−10.16, 2.76)	−0.5	Medium
	Chanco cheese	−2.10	(−10.02, 5.82)	−0.2	Small
	Oats with milk	−6.50	(−17.06, 4.06)	−0.6	Medium
	Couscous	−5.00	(−14.68, 4.67)	−0.5	Medium
	Buttery cheese	−7.90	(−19.46, 3.66)	−0.6	Medium
	Sliced bread	−5.50	(−15.86, 4.86)	−0.5	Medium
Q3	Lentils, 5 mm	−7.20	(−15.04, 0.64)	−0.8	Large
	Orange	−6.40	(−15.39, 2.60)	−0.6	Medium
	Tomato	−7.90	(−21.44, 5.64)	−0.5	Medium
	Cooked split peas	−8.00	(−18.33, 2.32)	−0.7	Large
	Short chicken drumstick	−7.40	(−19.50, 4.70)	−0.6	Medium
	Jack mackerel	−10.60	(−19.37, −1.83)	−1.1	Large
	Egg	−6.30	(−16.56, 3.96)	−0.6	Medium
	Seeded sliced bread	−6.30	(−20.71, 8.11)	−0.4	Medium
	Turkey breast	−10.90	(−20.44, −1.36)	−1.0	Large
Q4	Red apple	−7.40	(−22.83, 8.03)	−0.4	Medium
	Peanut	−14.40	(−25.60, −3.20)	−1.2	Large
	Cooked chickpeas	−13.60	(−27.91, 0.71)	−0.9	Large
	Brown rice	−13.00	(−28.66, 2.67)	−0.8	Large
	Beef	−8.60	(−22.34, 5.14)	−0.6	Medium
	Cooked beans	−14.80	(−28.41, 1.19)	−1.0	Large
	Lettuce	−17.30	(−31.82, −2.78)	−1.1	Large
	Raw beetroot	−3.30	(−19.27, 12.67)	−0.2	Small
	Carrot	−18.40	(−49.96, 13.16)	−0.5	Medium

**Table A3:** Mean differences, confidence intervals, and effect sizes (Hedges’ g) for normalized muscle activity ($\bar{V_{\%}}$) between the functional dentition (F) group and the complete denture wearer (D) group.

Quartile	Foods	$\bar{V_{\%}}$			
		F − D	CI	g	Effect Size
Q1	Yogurt	−0.15	(−0.27, −0.03)	−1.1	Large
	Yogurt with flaxseed	−0.12	(−0.25, 0.02)	−0.8	Large
	Yogurt with chia	−0.14	(−0.23, −0.04)	−1.3	Large
	PACAM cream	−0.13	(−0.24, −0.02)	−1.1	Large
	Ricotta	−0.11	(−0.25, 0.04)	−0.7	Large
	Peach jam	−0.11	(−0.22, −0.01)	−1.0	Large
	White cheese	−0.21	(−0.34, −0.09)	−1.6	Large
	Fruit cucumber	−0.21	(−0.34, −0.08)	−1.5	Large
	Avocado	−0.15	(−0.25, −0.05)	−1.4	Large
Q2	Salad cucumber	−0.15	(−0.31, 0.02)	−0.8	Large
	Kiwi	−0.24	(−0.41, −0.07)	−1.3	Large
	Lentils, 4 mm	−0.13	(−0.25, −0.00)	−0.9	Large
	Yellow banana	−0.25	(−0.43, −0.07)	−1.2	Large
	Chanco cheese	−0.23	(−0.41, −0.05)	−1.1	Large
	Oats with milk	−0.05	(−0.19, 0.09)	−0.3	Medium
	Couscous	−0.14	(−0.23, −0.04)	−1.3	Large
	Buttery cheese	−0.18	(−0.35, −0.01)	−1.0	Large
	Sliced bread	−0.14	(−0.25, −0.04)	−1.2	Large
Q3	Lentils, 5 mm	−0.13	(−0.24, −0.02)	−1.1	Large
	Orange	−0.17	(−0.35, 0.02)	−0.8	Large
	Tomato	−0.28	(−0.47, −0.10)	−1.4	Large
	Cooked split peas	−0.14	(−0.25, −0.03)	−1.2	Large
	Short chicken drumstick	−0.04	(−0.16, 0.07)	−0.3	Medium
	Jack mackerel	−0.06	(−0.18, 0.05)	−0.5	Medium
	Egg	−0.17	(−0.28, −0.07)	−1.5	Large
	Seeded sliced bread	−0.11	(−0.20, −0.02)	−1.1	Large
	Turkey breast	−0.07	(−0.2, 0.05)	−0.5	Medium
Q4	Red apple	−0.19	(−0.43, 0.05)	−0.7	Large
	Peanut	−0.01	(−0.23, 0.08)	−0.5	Medim
	Cooked chickpeas	−0.13	(−0.25, −0.01)	−0.9	Large
	Brown rice	−0.14	(−0.25, −0.03)	−1.2	Large
	Beef	−0.09	(−0.22, 0.04)	−0.6	Medium
	Cooked beans	−0.12	(−0.24, −0.01)	−1.0	Large
	Lettuce	−0.18	(−0.40, 0.04)	−0.7	Large
	Raw beetroot	−0.27	(−0.55, 0.01)	−0.9	Large
	Carrot	−0.17	(−0.40, 0.06)	−0.7	Large

**Table A4:** Mean differences, confidence intervals, and effect sizes (Hedges’ g) for the chewing index (*Ci*) between the functional dentition (F) group and the complete denture wearer (D) group.

Quartile	Foods	*Ci*			
		F − D	CI	g	Effect Size
Q1	Yogurt	−0.14	(−0.29, 0.01)	−0.9	Large
	Yogurt with flaxseed	−0.27	(−1.19, 0.65)	−0.3	Medium
	Yogurt with chia	−0.45	(−1.30, 0.40)	0.5	Medium
	PACAM cream	−0.85	(−2.79, 1.08)	−0.4	Medium
	Ricotta	−0.26	(−1.72, 1.20)	−0.2	Small
	Peach jam	−0.52	(−2.26, 1.21)	−0.3	Medium
	White cheese	−5.23	(−10.76, 0.30)	−0.9	Large
	Fruit cucumber	−4.23	(−10.53, 2.08)	−0.6	Medium
	Avocado	−14.40	(−35.50, 6.70)	−0.6	Medium
Q2	Salad cucumber	−9.00	(−24.39, 6.37)	−0.5	Medium
	Kiwi	−14.35	(−23.87, −4.82)	−1.4	Large
	Lentils, 4 mm	−9.26	(−23.63, 5.12)	−0.6	Medium
	Yellow banana	−24.46	(−42.88, −6.05)	−1.2	Large
	Chanco cheese	−23.53	(−49.85, 2.77)	−0.8	Large
	Oats with milk	−30.49	(−76.87, 15.91)	−0.6	Medium
	Couscous	−28.86	(−64.25, 6.53)	−0.7	Large
	Buttery cheese	−37.57	(−78.02, 2.88)	−0.8	Large
	Sliced bread	−33.50	(−74.26, 7.26)	−0.7	Large
Q3	Lentils, 5 mm	−35.52	(−73.21, 2.17)	−0.8	Large
	Orange	−39.40	(−79.77, 0.98)	−0.9	Large
	Tomato	−45.11	(−103.05, 12.83)	−0.7	Large
	Cooked split peas	−39.59	(−67.72, −11.45)	−1.3	Large
	Short chicken drumstick	−44.70	(−96.53, 7.13)	−0.8	Large
	Jack mackerel	−47.40	(−86.70, −8.10)	−1.1	Large
	Egg	−47.75	(−96.44, 0.93)	−0.9	Large
	Seeded sliced bread	−45.04	(−114.97, 24.88)	−0.6	Medium
	Turkey breast	−54.83	(−95.53, −14.13)	−1.2	Large
Q4	Red apple	−48.36	(−125.97, 29.24)	−0.6	Medium
	Peanut	−78.21	(−122.90, −33.50)	−1.8	Large
	Cooked chickpeas	−96.50	(−194.34, 1.35)	−0.9	Large
	Brown rice	−112.91	(−217.06, −8.76)	−1.0	Large
	Beef	−79.37	(−159.92, 1.07)	−0.9	Large
	Cooked beans	−133.84	(−227.01, −40.67)	−1.3	Large
	Lettuce	−143.66	(−247.92, −39.40)	−1.2	Large
	Raw beetroot	−149.83	(−315.81, 16.16)	−0.9	Large
	Carrot	−210.61	(−464.95, 43.74)	−0.7	Large
